# Microtubule Integrity Is Associated with the Functional Activity of Mitochondria in HEK293

**DOI:** 10.3390/cells10123600

**Published:** 2021-12-20

**Authors:** Min Jeong Cho, Yu Jin Kim, Won Dong Yu, You Shin Kim, Jae Ho Lee

**Affiliations:** 1 Department of Biomedical Science, College of Life Science, CHA University, Pocheon 11160, Korea; mjjj0725@gmail.com (M.J.C.); manseo60@gmail.com (W.D.Y.); 2 Laboratory of Reproductive and Molecular Medicine, CHA Fertility Center Seoul, Jung-gu, Seoul 04637, Korea; yj_kim@chamc.co.kr; 3 Department of Obstetrics and Gynecology, CHA Fertility Center Seoul Station, Seoul 04637, Korea; 4 Department of Obstetrics and Gynecology, CHA University School of Medicine, Seoul 04637, Korea

**Keywords:** mitochondria, microtubule, stability, disturber, bioenergetic activity

## Abstract

Mitochondria move along the microtubule network and produce bioenergy in the cell. However, there is no report of a relationship between bioenergetic activity of mitochondria and microtubule stability in mammalian cells. This study aimed to investigate this relationship. We treated HEK293 cells with microtubule stabilizers (Taxol and Epothilone D) or a microtubule disturber (vinorelbine), and performed live-cell imaging to determine whether mitochondrial morphology and bioenergetic activity depend on the microtubule status. Treatment with microtubule stabilizers enhanced the staining intensity of microtubules, significantly increased ATP production and the spare respiratory capacity, dramatically increased mitochondrial fusion, and promoted dynamic movement of mitochondria. By contrast, bioenergetic activity of mitochondria was significantly decreased in cells treated with the microtubule disturber. Our data suggest that microtubule stability promotes mitochondrial functional activity. In conclusion, a microtubule stabilizer can possibly recover mitochondrial functional activity in cells with unstable microtubules.

## 1. Introduction

Mitochondrial metabolism is crucial for bioenergy production [1]. The quantity and quality of mitochondria and their impact on the cell are related to several chronic diseases, such as obesity, diabetes, and vision loss [2,3,4,5]. However, factors that can restore mitochondrial function have not been clearly identified for clinical therapeutic applications [2]. Mitochondrial dysfunctional processes are not completely understood [2]. Diverse factors involved in other biochemical pathways that affect mitochondria must be studied. Mitochondria display macroscopic behaviors (termed dynamics) including fusion, fission, transport, and mitophagy [6,7]. Although these behaviors are molecularly distinct from bioenergetic reactions, recent studies suggest that they are tightly linked and regulate one another [1]. Microtubules are a cytoskeletal component, and are critical for cell morphology and normal organelle function and vesicle trafficking [8]. The stability of microtubules is associated with degenerative diseases such as dementia and aging [9,10,11]. The integrity of microtubules is important for dynamic movement of mitochondria in the cell. In neurons, mitochondria mostly move from soma to axons to provide energy throughout these cells [12]. Mitochondria supply energy to dynamic microtubules and maintain microtubule integrity to support cellular organization, which is important for cellular behaviors such as proliferation and migration [13]. Microtubule-associated proteins such as Tau regulate microtubule integrity [9,12,14]. Tau is a biomarker of dementia and a therapeutic target protein for neurodegenerative disorders [9]. Bioenergy produced by mitochondria supports assembly of microtubule-associated proteins for microtubule polymerization during cell growth and migration. Microtubule stabilizers such as Taxol and Epothilone D (EpD) play a similar role to microtubule-associated proteins and inhibit microtubule disassembly-dependent chromosome segregation, and thus can be used as anti-cancer therapies [15,16,17]. Anti-cancer agents such as microtubule stabilizers regulate mitochondrial structure, movement, and function in cancer cells, and thereby induce apoptosis [18,19,20]. In addition, microtubule stabilizers can be used to prevent microtubule destabilization in neurons and facilitate transport of vesicles and mitochondria for treatment of neurodegeneration [21]. We previously reported that microtubule instability significantly decreases bioenergetic activity of mitochondria in mouse oocytes [22]. However, the relationship between microtubule stability and bioenergetic activity of mitochondria has not been clearly demonstrated. Mitochondria play a major role in supporting life by synthesizing building blocks such as amino acids, lipids, and nucleotides, and by facilitating cell growth through catabolism and anabolism of nutrients. Several key factors such as the serine/threonine kinase AMP-activated protein kinase (AMPK) and mechanistic target of rapamycin (mTOR) regulate mitochondrial metabolism [23,24,25]. These factors are associated with multiple metabolic signaling pathways that underlie the functional activity of mitochondria. AMPK senses low levels of ATP to enhance cellular energy metabolism; it is also the main regulator of responses to cellular energetic stress and mitochondrial insults, and coordinates multiple features of autophagy and mitochondrial biology [23]. The roles of mTOR include integration of proteins for homeostasis, and synthesis of proteins for cell growth and survival [25]. mTOR also regulates assembly of microtubule proteins and restores cell proliferation. Loss-of-function of AMPK and mTOR causes age-related diseases such as neuronal degeneration, cardiac disease, and cancer [23,25]. The relationship between microtubule integrity and the functional activity of mitochondria in mammalian cells, and the involvement of AMPK and mTOR in this relationship, are unclear.

Here, we hypothesized that microtubule stability is linked to bioenergy production of mitochondria in mammalian cells. We treated cells with microtubule stabilizers or a microtubule disturber and demonstrated that microtubule stability is associated with bioenergetic activity of mitochondria in mammalian cells.

## 2. Materials and Methods

### 2.1. Preparation of HEK293 Cells

HEK293 cells were cultured in Dulbecco’s minimum essential medium (DMEM; SH30243.01; Hyclone, UT, USA) supplemented with 10% fetal bovine serum (16000-044; Gibco, CA, USA) and 1% penicillin/streptomycin (15140122, Gibco), according to a general cell culture protocol. A total of 4 × 10^5^ cells were seeded on a cell culture dish (90 × 20 mm; SPL-20100; SPL, Pocheon, Korea) and kept in an incubator containing 5% CO_2_ at 37 °C. Cells were passaged every 3–4 days when they reached 70–80% confluency. At every passage, the culture medium was changed 2 days after seeding.

### 2.2. Treated Microtubule Stabilizer and Disturber to HEK293 Cells

The microtubule stabilizers Taxol (PHL89806; Sigma-Aldrich, CA, USA) and EpD (ab143616; Abcam, Cambridge, UK), and the microtubule disturber vinorelbine (VNB; V2264-5MG, Sigma-Aldrich), were used. The optimal concentrations of these chemical agents were determined in HEK293 cells. Cells were seeded at a density of 1 × 10^4^ cells (100 µL) per well in a 96-well plate (SPL). The next day, cells were treated with Taxol (1, 10, 100, and 1000 nM), EpD (2, 20, 200, and 2000 pM), and VNB (1, 10, 100, and 1000 nM) overnight, and then cell proliferation was investigated using a WST-8 Cell Viability Assay Kit (QM2500; Biomax, Seoul, Korea). This assay assesses the cell number by measuring the reaction between WST-8 and electrons released from NADH, enabling calculation of the proliferation rate. In total, 10 μL WST-8 solution was added to the cell-containing medium at Days 0, 3 and 6, according to the manufacturer’s protocol. After allowing the reaction to proceed for 1 h, absorbance at 450 nm was measured using a microplate spectrometer (Epoch™ Microplate Spectrophotometer; BioTek, VT, USA).

### 2.3. Measurement of the Oxygen Consumption Rate (OCR)

The OCR was measured using a Seahorse XFp Analyzer (Agilent, CA, USA). A total of 10,000 cells/well were seeded into a Seahorse XFp Cell Culture Miniplate, incubated for 24 h, and then cultured overnight at 37 °C with each microtubule stabilizer (1 nM Taxol or 2 pM EpD) or the microtubule disturber (10 nM VNB). Thereafter, each sample plate was switched to Seahorse XF Base Medium containing 2 mM L-glutamine, 5.5 mM D-glucose, and 1 mM sodium pyruvate, according to the composition of DMEM. The OCR was measured for 2 min following mixing for 3 min. Cells were treated with 1 µM oligomycin, 0.5 µM FCCP, and 0.5 µM rotenone/antimycin A. The basal OCR was normalized according to the protein concentration determined by the BCA assay. The results obtained with four samples of HEK293 cells were normalized, and the basal respiration rate, ATP production, proton leak, and coupling efficiency were simultaneously calculated using the manufacturer’s software Multi-File_Seahorse XF Cell Mito Stress Test Report Generator. A stacked vertical bar graph of mitochondrial profile values was also plotted using SigmaPlot version 12.5 software.

### 2.4. Immunocytochemistry to Assess Microtubule Formation

For immunocytochemistry, 1 × 10^3^ control, 1 nM Taxol-treated, 2 pM EpD-treated, and 10 nM VNB-treated HEK293 cells were seeded on glass bottom dishes and cultured overnight at 37 °C. Cells were fixed with cold methanol for 5 min at −80 °C, washed with phosphate-buffered saline (PBS), and incubated overnight at 4 °C with a mouse anti-alpha-tubulin monoclonal antibody (12G10; DSHB, Iowa City, IA, USA) diluted 1:100 in PBS containing 0.1% bovine serum albumin. Thereafter, the samples were washed, incubated with an anti-mouse Alexa Fluor 555-conjugated secondary antibody for 2 h at room temperature, and washed with PBS. Nuclei were stained with 1 µg/mL Hoechst^®^ 33342 (H1399; Thermo Fisher Life Technologies, California, CA, USA), and F-actin was stained with Alexa Fluor 488^®^ phalloidin (A12379; Thermo Fisher Scientific, CA, USA) for 5 min at room temperature. After three washes with PBS, stained cells were mounted on glass slides with anti-fade mounting medium (VECTASHIELD H-1000; Vector Laboratories, Burlingame, CA, USA). Images of stained cells were acquired using a LSM880 confocal microscope equipped with Airyscan META (Carl Zeiss AG, Oberkochen, Germany). Z-stacks of 15 images were analyzed using ZEN2010 software (Carl Zeiss AG). Each Z-stack image was produced using maximum intensity projection by Zeiss software (ZEN 2012 version 8; Carl Zeiss AG, Oberkochen, Germany).

### 2.5. Investigation of Mitochondrial Functional Activity by Confocal Microscopy

The mitochondrial membrane potential and mitochondrial mass of individual cells were determined using MitoSpy Orange CMTMRos (424803; BioLegend, San Diego, CA, USA), which is useful to indicate cell health and mitochondrial localization, and MitoSpy Green FM (424805, BioLegend), respectively. HEK293 cells (2 × 10^4^/mL) were passaged on glass bottom confocal dishes (30012, SPL), treated with microtubule stabilizers (1 nM Taxol and 2 pM EpD) or a microtubule disturber (10 nM VNB) for 16 h; incubated in DMEM containing 250 nM MitoSpy Green FM and 250 nM MitoSpy Orange CMTMRos for 5 min; and washed with culture medium. Then, nuclei were stained with 1 µg/mL Hoechst 33342^®^ (H1399, Thermo Fisher Life Technologies) for 5 min at room temperature. Finally, samples were imaged using a confocal microscope with a live-cell chamber system (LSM880, Carl Zeiss AG). For time-lapse live-cell imaging, 120 images were captured at a time interval of 0.5 s. Each image was analyzed and exported as a moving file (25 frames/s) and a single picture (TIFF format) using ZEN2012 software (Carl Zeiss AG). Images were evaluated using ImageJ (version 1.08) with the Difference Tracker plug-in. The movies were used to calculate the average percentage of mitochondrial intensity moving and maximum speed of mitochondria in the cytoplasm.

### 2.6. Gene Expression Analysis

Total RNA was isolated from HEK293 cells using TRIzol according to the manufacturer’s procedures (15596026; Invitrogen, Illinois, IL, USA). A total of 100 ng total RNA was transcribed into cDNA using AccuPower^®^ CycleScript RT PreMix (K2050; Bioneer, Daejeon, Korea), which was then amplified with AccuPower^®^ Taq PCR PreMix (Bioneer) using primers specific to human MFN1, MFN2, OPA1, DRP1, and glyceraldehyde 3-phosphate dehydrogenase (GAPDH) (Supplement Table S1). A total of 10 pmol/µL forward and reverse primers, and 100 ng template cDNA, were added to AccuPower^®^ Taq PCR PreMix tubes, and nuclease-free water (AM9930; Ambion, Texas, TX, USA) was added up to a total volume of 20 µL. The PCR cycling conditions were as follows: 1 min at 95 °C, followed by 30 cycles of denaturation for 30 s at 95 °C, annealing for 30 s at 60 °C, and extension for 50 s at 72 °C. PCR products were resolved on 1.5% agarose gels with Safeview™ (Applied Biological Materials, British Columbia, BC, Canada). The gels were visualized under ultraviolet illumination using a gel documentation system (WSE-6100 LuminoGraph; ATTO, Tokyo, Japan). Real-time qPCR was also performed using SsoAdvanced Universal SYBR Green Supermix (#1725270; Bio-Rad, California, CA, USA) on a spectro-fluorometric thermal cycler (CFX96 Touch Real-Time PCR Detection System, Bio-Rad). The reaction contained 10 pmol/µL forward and reverse primers, 200 ng template cDNA, and 2× SsoAdvanced Universal SYBR Green Supermix. Distilled water was added up to a final volume of 20 µL. The PCR cycling conditions were as follows: 3 min at 95 °C, followed by 40 cycles of denaturation for 10 s at 95 °C, and annealing and extension for 20 s at 60 °C. The expression of each examined gene was normalized to that of GAPDH. Triplicate samples were tested.

### 2.7. Protein Expression Analysis

Trypsinized HEK293 cells were immediately harvested, centrifuged, and washed with PBS without Ca2+ and Mg2+ (LB001-02, Hyclone). All samples were stored at −80 °C until western blot analysis. Proteins were extracted from each sample with Pro-Prep protein lysis buffer (17081; iNTron, Seongnam, Korea) and quantified with a protein quantification assay kit (BCA assay kit, BCA500; Biomax). Samples were then boiled in 4× Laemmli sample buffer (1610747, Bio-Rad) containing 2-mercaptoethanol (161-0710, Bio-Rad) for 5 min. The same amount of protein was loaded into each well of an 8% sodium dodecyl sulfate polyacrylamide gel. Gels were electrophoresed at 60 V for 30 min and then at 120 V for 1 h. The proteins were transblotted onto a nitrocellulose membrane (BR162-0112, Bio-Rad) at 100 V for 70 min. The membrane was incubated with blocking buffer (TBS-T containing 5% bovine serum albumin) for 1 h and then with a primary antibody solution overnight at 4 °C. Mouse anti-AMPK (AHO1332; Thermo Fisher, IL, USA), rabbit anti-pAMPK (701068; Thermo Fisher Scientific, CA, USA), rabbit anti-mTOR (2983T; Cell Signaling, Massachusetts, MA, USA), rabbit anti-pmTOR (ab109268, Abcam), and mouse anti-GAPDH (MA5-15738, Invitrogen) primary antibodies were used. The blotted membrane was washed with TBS-T, and incubated with horseradish peroxidase-conjugated anti-mouse (G-21040, Invitrogen) and anti-rabbit (31460, Invitrogen) secondary antibodies for 90 min at room temperature. Immunoreactive bands were detected using an enhanced chemiluminescence detection reagent (ClarityTM western blot substrate; 1705061, Bio-Rad). Images of the bands were acquired using ImageSaver version 6 (ATTO). The intensity of each band was analyzed with CA4 analyzer software (version 2.3.1, ATTO). The band intensities were normalized against that of GAPDH. The experiment was repeated three times under the same conditions with different samples.

### 2.8. Statistical Analysis

All data are expressed as means ± standard error of the mean (SEM) of triplicate measurements. Statistical analyses were carried out using a one-way analysis of variance (ANOVA) with the Bonferroni test of variance. Significant differences are indicated by asterisks (* *p* < 0.05, ** *p* < 0.01, and *** *p* < 0.001) in the figures.

## 3. Results

### 3.1. Morphology, Viability, and Proliferation of Microtubule Stabilizer- and Microtubule Disturber-Treated Cellsr

**Figure 1:**
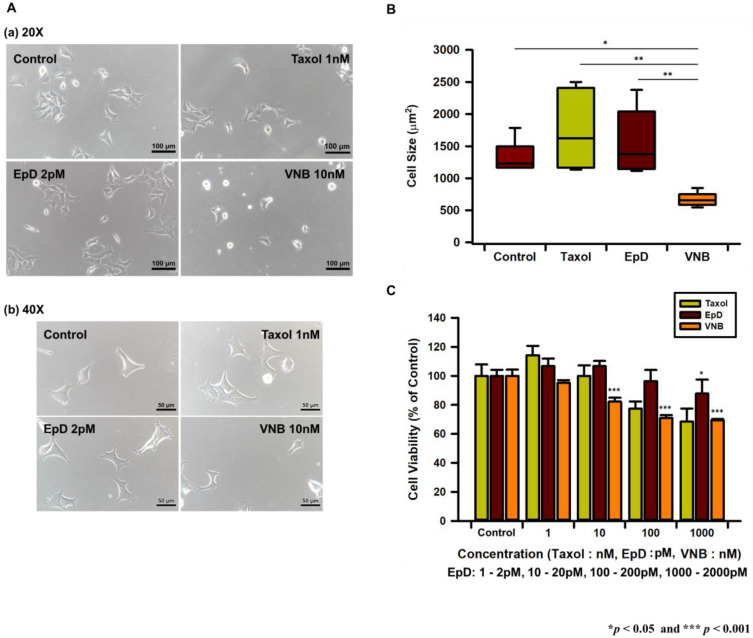

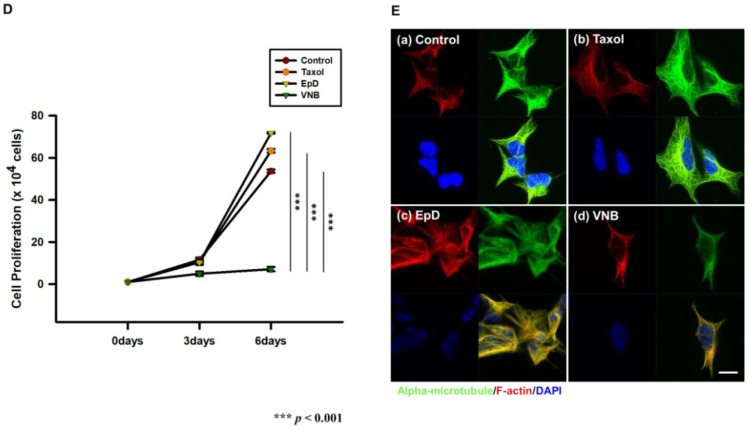
Microscope images, viability, and proliferation of microtubule stabilizer- and microtubule disturber-treated cells. (**A**) Light microscope imaging of cells treated with Taxol, EpD, and VNB. (**a**) Image of 20×; (**b**) image of 40×; (**B**) graph of cell size following treatment with Taxol, EpD, and VNB. (**C**) Graph of cell viability following treatment with Taxol, EpD, and VNB. (**D**) Graph of cell proliferation at 3 and 6 days after treatment with Taxol, EpD, and VNB. (**E**) Confocal images of microtubules and F-actin in (**a**) control, (**b**) Taxol-treated, (**c**) EpD-treated, and (**d**) VNB-treated cells. Scale bar = 20 μm. The data are presented as means ± SEM of three replicates. Significant differences are indicated by asterisks (* *p* < 0.05, ** *p* < 0.01, *** *p* < 0.001.). Statistical significance was determined by a one-way ANOVA.

We treated HEK293 cells with the microtubule stabilizers Taxol and EpD and the microtubule disturber VNB. Microtubule stabilizer-treated cells were well spread and measured about 1400 μm^2^, while VNB-treated cells were 2-fold smaller than control and microtubule stabilizer-treated cells (Figure 1A,B).

We evaluated cell viability and proliferation. Viability of microtubule stabilizer-treated cells was significantly higher than that of 10–1000 nM VNB-treated cells after treatment overnight with each reagent (Figure 1C). Treatment with 1 nM Taxol and 2 pM EpD increased cell viability compared with the control. Viability of VNB-treated cells was significantly lower than that of control and microtubule stabilizer-treated cells (Figure 1D). After 6 days, proliferation of microtubule stabilizer-treated cells was significantly higher than that of VNB-treated cells and was higher than that of control cells (Figure 1D).

We analyzed the arrangement and formation of microtubules by performing immunocytochemistry with an anti-alpha-tubulin antibody and phalloidin, which labels microtubules and F-actin (Figure 1E). Microtubule staining was stronger in cells treated with microtubule stabilizer than cells treated with control and VNB-treated. Treatment with Taxol or EpD increased microtubule thickness. Treatment with VNB significantly decreased cell viability, and the VNB-treated cells that survived mostly contained thin and fragmented microtubules in comparison with Taxol- and EpD-treated cells. F-actin fibers were less dense and thinner in VNB-treated cells. Microtubules were denser and thicker in Taxol- and EpD-treated cells than in VNB-treated cells.

### 3.2. Microtubule Stabilizer Treatment Increases Mitochondrial Oxygen Consumption and ATP Production

**Figure 2:**
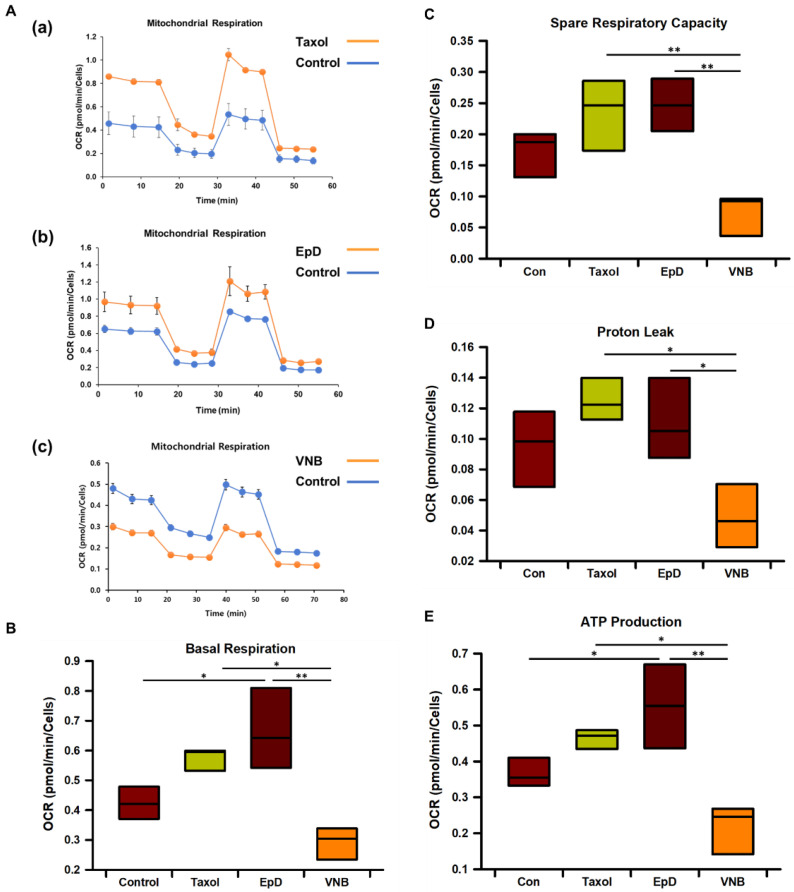
Analysis of mitochondrial respiration in microtubule stabilizer- and microtubule disturber-treated cells. (**A**) Real-time mitochondrial respiration, indicated by the OCR, in (**a**) Taxol-treated, (**b**) EpD-treated cells, and (**c**) VNB-treated cells (orange line), as well as control cells (blue line), measured using an extracellular flux analyzer. (**B**) Basal respiration, (**C**) the spare respiratory capacity, (**D**) proton leak, and (**E**) ATP production based on the OCR in cells treated with Taxol, EpD, and VNB measured using an extracellular flux analyzer. Data were automatically generated by the manufacturer’s software and are presented as means ± SEM (*n* = 6 for control, Taxol-treated, EpD-treated, and VNB-treated cells). Statistical significance was determined by a one-way ANOVA. * *p* < 0.05, ** *p* < 0.01.

Next, we investigated mitochondrial functional activity. The level of mitochondrial respiration was determined by measuring the OCR in HEK293 cells (Figure 2A). Treatment with a microtubule stabilizer increased oxygen consumption and ATP production. Therefore, we investigated mitochondrial metabolism using an extracellular flux analyzer, which simultaneously determined several metabolic profiles including basal respiration, proton leak, and the spare respiratory capacity (SRC). Basal respiration was ~0.6–0.7 pmol/min/cell higher in microtubule stabilizer-treated cells than in control cells, but was ~0.3 pmol/min/cell lower in VNB-treated cells than in control cells (Figure 2B). The SRC, an indicator of healthy mitochondria, was significantly higher in Taxol- and EpD-treated cells than in VNB-treated cells (Figure 2C). In addition, the SRC was higher in Taxol- and EpD-treated cells than in control cells, but this difference was not statistically significant. Proton leak was significantly higher in Taxol- and EpD-treated cells than in VNB-treated cells (Figure 2D). ATP production was significantly higher in Taxol- and EpD-treated cells than in control cells, but was decreased in VNB-treated cells. The coupling efficiency, which is the percentage of mitochondrial respiration linked to ATP production, was nearly equal in the two groups of Taxol, EpD treated cells (Figure 2E). These results suggest that microtubule stabilizer treatment enhances mitochondrial metabolism by increasing oxygen consumption.

### 3.3. Dynamic Properties of Mitochondria in Microtubule Stabilizer- and Microtubule Disturber-Treated Cells

**Figure 3:**
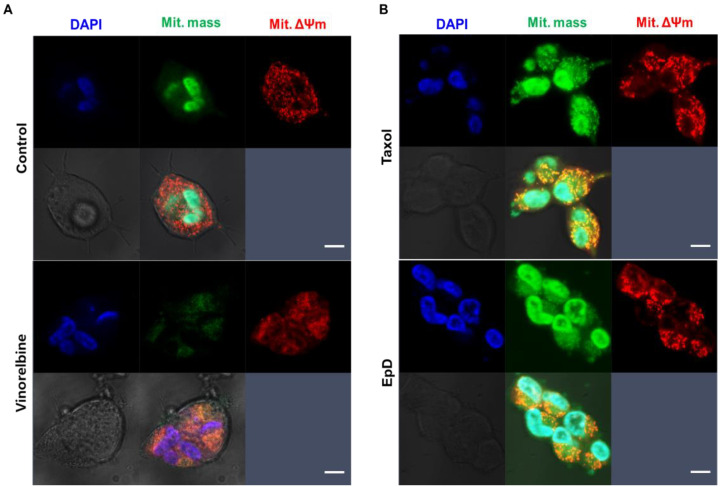
Confocal live-cell imaging to analyze mitochondrial mass and the mitochondrial membrane potential in microtubule stabilizer- and microtubule disturber-treated cells. Representative confocal images of (**A**) control and VNB-treated cells, and (**B**) Taxol- and EpD-treated cells. Mitochondrial mass, mitochondrial membrane potential (ΔΨm), and nuclei are represented in green, red, and blue, respectively. Scale bar = 20 μm.

We performed live-cell confocal imaging to analyze the mass and membrane potential of mitochondria (Figure 3). The level of green fluorescence, indicative of mitochondrial mass, was markedly higher in microtubule stabilizer-treated cells (Figure 3B) than in control and microtubule disturber-treated cells (Figure 3A). In microtubule stabilizer-treated cells, the shape of mitochondria was dramatically changed such that they became large and oval, indicative of mitochondrial fusion (Figure 3B). By contrast, in microtubule disturber-treated cells, mitochondria were rod-shaped, indicative of mitochondrial fission (Figure 3A).

**Figure 4:**
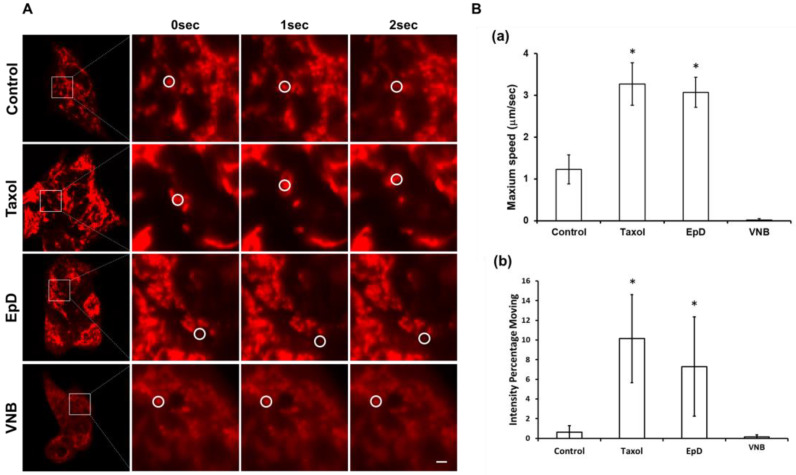
Confocal live-cell imaging to the tracking of mitochondria movement in microtubule stabilizer- and microtubule disturber-treated cells. (**A**) Confocal live-cell imaging of control, Taxol-treated, EpD-treated, and VNB-treated cells. White circles indicate the localization of mitochondria. Scar bar = 2 μM. (**B**) Graphs of the (**a**) maximum speed of mitochondria and (**b**) percentage of moving mitochondria in control, Taxol-treated, EpD-treated, and VNB-treated cells. Data are presented as means ± SEM of three replicates. Significant differences are indicated by asterisks (* *p* < 0.05).

We performed live-cell confocal imaging to analyze the movement of mitochondria (Figure 4). The level of microtubule staining was higher in microtubule stabilizer-treated cells than in control and microtubule disturber-treated cells (Figure 4A). Movement of mitochondria was three times faster in microtubule stabilizer-treated cells than in control cells (Figure 4B; Supplemental Video S1; B: EpD-treated cells, C: Taxol-treated cells). The maximum speed of mitochondrial movement was 3 µm/s in microtubule stabilizer-treated cells (Supplemental Video S1; B: Taxol-treated cells, C: EpD-treated cells) and 1 µm/s in control cells (Figure 4B). However, mitochondria barely moved in microtubule disturber-treated cells compared with microtubule stabilizer-treated cells (Supplemental Video S1; A: control cells, D: VNB-treated cells). We also analyzed the average percentage of moving mitochondrial fluorescence staining in each experimental condition, which represents the percentage of mitochondria movement in each cell.

**Figure 5:**
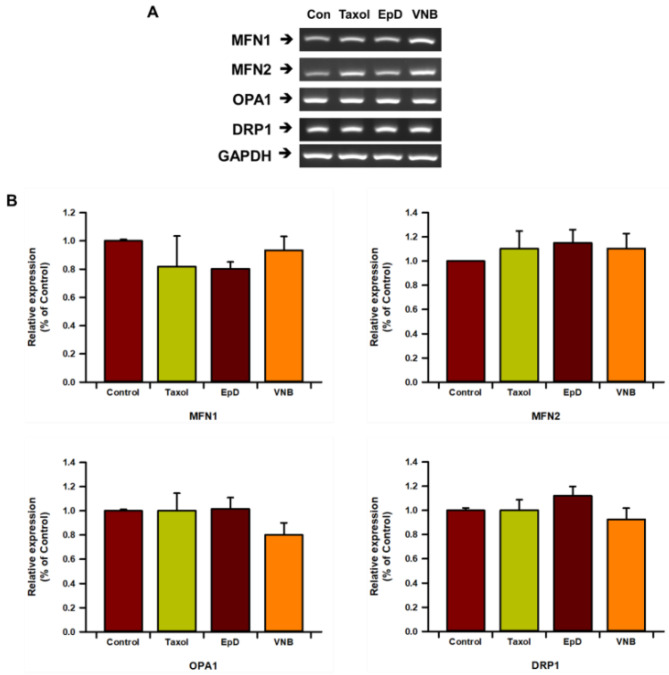
Gene expression analysis of MFN1, MFN2, OPA1, and DRP1 in microtubule stabilizer- and microtubule disturber-treated cells. (**A**) RT-PCR analysis. (**B**) Real-time qPCR analysis. Data are presented as means ± SEM of three replicates.

Next, we analyzed mRNA expression of the mitochondrial fusion factors MFN1, MFN2, and OPA1, and of the mitochondrial fission factor DRP1, using RT-PCR (Figure 5A) and real-time qPCR (Figure 5B). mRNA expression of DRP1 was increased in microtubule stabilizer-treated cells and was lower in VNB-treated cells than in Taxol- and EpD-treated cells, but none of these differences were significant. mRNA expression of MFN1 and MFN2 exhibited the reverse pattern to that of DRP1, but there were no significant differences.

### 3.4. Treatment with a Microtubule Stabilizer Activates the mTOR Signaling Pathway, Which Is a Critical Regulator of Mitochondrial Metabolic Activity

**Figure 6:**
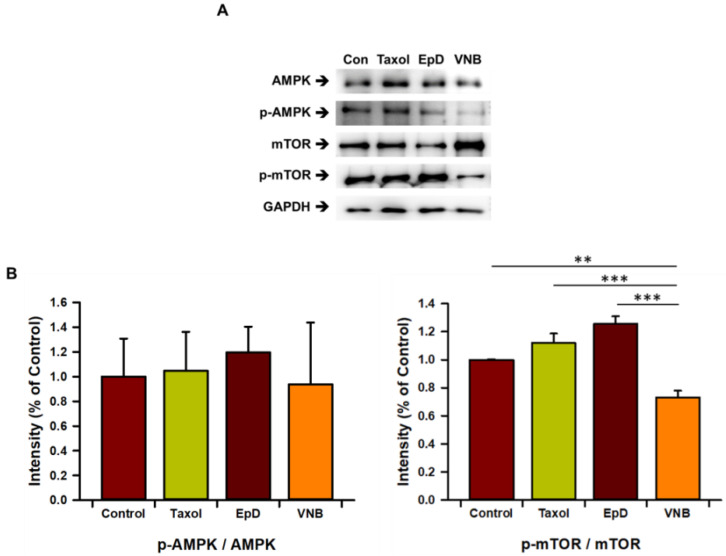
Protein expression of AMPK and mTOR in microtubule stabilizer- and microtubule disturber-treated cells. (**A**) Blots of AMPK, pAMPK, mTOR, and pmTOR in control, Taxol-treated, EpD-treated, and VNB-treated cells. (**B**) Graphs of the band intensities of pAMPK/AMPK and pmTOR/mTOR in control, Taxol-treated, EpD-treated, and VNB-treated cells. The data are presented as means ± SEM of three replicates. Significant differences are indicated by asterisks (** *p* < 0.01, *** *p* < 0.001).

We analyzed the expression of AMPK, mTOR (a main component of mTORC1), and their phosphorylated (i.e., active) forms by Western blotting (Figure 6). Treatment with a microtubule stabilizer enhanced phosphorylation of mTOR in HEK293 cells. The pAMPK/AMPK ratio was slightly lower in VNB-treated cells than in Taxol- and EpD-treated cells; however, there were no significant differences between the groups (Figure 6A). The pmTOR/mTOR ratio was significantly higher in Taxol-treated, EpD-treated, and control cells than in VNB-treated cells (Figure 6B). These results show that Taxol and EpD increase phosphorylation of mTOR, which is a central energy regulator in HEK293 cells and part of a major protein complex involved in cellular physiology.

## 4. Discussion

In this study, we found that microtubule stability is associated with the functional activity of mitochondria. Bioenergy production was significantly increased in cells treated with Taxol or EpD, which are microtubule stabilizers, but was dramatically decreased in cells treated with the microtubule disturber VNB. These findings demonstrate that there is an association between microtubule stability and mitochondrial functional activity, which is related to ATP production by oxidative phosphorylation in mitochondria, in HEK293 cells. Treatment with a microtubule stabilizer significantly increased mitochondrial ATP production and the SRC of mitochondria, whereas microtubule disturber (VNB)-treated cells exhibited substantial decreases in mitochondrial motility and ATP production. Microtubules were unstable in VNB-treated cells. Therefore, microtubule stability may affect mitochondrial metabolic activity. These data suggest the new concept that microtubule stability is involved in regulation of mitochondrial properties including migration and proliferation, contrary to commonly held views regarding mitochondrial bioenergy production in mainstream biology. In summary, our data demonstrate that microtubule stability enhances bioenergetic activity of mitochondria.

We used the microtubule stabilizers Taxol and EpD. Microtubule-targeted agents are used for anti-cancer therapy. The mechanism of action of anti-cancer agents such as microtubule stabilizers has not been completely elucidated, but there are reports that microtubule-targeted agents affect the apoptotic signaling pathway through mitochondria [20]. However, these agents have been reported to have different effects according to the dose used. In a preclinical study, EpD increased the number of microtubules and reduced the number of axons with an abnormal morphology in young and aged Tau-transgenic mice [26]. In other mouse models of tauopathy, EpD improved cognition and reduced Tau pathology and Tau-related changes in microtubule dynamics [15]. Among the many microtubule-directed drugs, members of the Taxol family are unique because they stabilize microtubules against depolymerization [10]. This reduces stochastic switching of microtubules between growth and shrinkage, a classical behavior known as “dynamic assembly,” which is essential for normal functioning of the microtubule cytoskeleton. The integrity of microtubules is correlated with several biological properties, such as cell signaling and organelle trafficking [27]. The microtubule network is required for intracellular movement of mitochondria and is related to mitochondrial quality control [28]. Microtubule rearrangement supports mitochondrial bioenergy which affects cell proliferation [29]. However, there are no reports that microtubule stability promotes the functional activity of mitochondria. Our data demonstrate that microtubule stability enhances bioenergy production and dynamic activation of mitochondria, and increases cell viability and proliferation.

The OCR was higher in microtubule stabilizer-treated cells than in control cells. The OCR is an established measure of mitochondrial function and an indicator of normal cellular function [30]. Basal respiration, proton leak, non-mitochondrial respiration, ATP-linked respiration, the SRC, and maximal respiration were investigated. Proton leak is increased, and basal and maximal respiration are decreased, in dysfunctional mitochondria. Basal respiration was higher in Taxol- and EpD-treated cells than in control cells, but clearly decreased in VNB-treated cells, demonstrating that there was an increase in dysfunctional mitochondria in the latter cells. The SRC was higher in microtubule stabilizer-treated cells than in control and VNB-treated cells. The high SRC demonstrates that optimal energy persists in mitochondria in microtubule stabilizer-treated cells, meaning that treatment with a microtubule stabilizer increased the population of healthy mitochondria.

Mitochondrial mass is a quantitative marker of mitochondria and is associated with cell survival and a protective effect via activation of mitochondrial biogenesis [1]. Mitochondrial mass was much higher in microtubule stabilizer-treated cells than in control and VNB-treated cells. The mitochondrial membrane potential is also linked to cell survival, which is blockage of respiration coupled with the inner membrane [31,32]. It plays a critical role in mitochondrial homeostasis and selective elimination of dysfunctional mitochondria. Excessive elevation of the mitochondrial membrane potential stimulates cell death, i.e., apoptosis and necroptosis, which cause several diseases [32]. The dynamic activities of mitochondria have several roles depending on the cell type and status [6,33]. Microtubule stability is correlated with mitochondrial dynamics such as fusion, fission, and transport [34]. Our data show that microtubule stabilizers induced fusion (oval shape) of mitochondria and increased movement of mitochondria for bioenergy supply, and increased cell proliferation. Fusion of mitochondria enables mitochondrial content exchange and calcium and reactive oxygen species buffering, which promote overall mitochondrial function [33]. Mitochondrial fission is essential for cell growth and division because it provides sufficient mitochondria, sustains cell polarity, and helps to eliminate damaged mitochondria [35]. The present study revealed that treatment with a microtubule disturber disrupted microtubule integrity, delayed cell proliferation, and increased fission of mitochondria. Mitochondria fusion is indicative of a healthy mitochondrial population and is required to maintain mitochondrial function. It has essential roles in the maintenance of active mitochondria. Mitochondrial fission and fusion correlate with bioenergetic activity and are related to aging and age-related diseases [7,36]. However, the mechanism of fission and fusion of mitochondria remain unclear.

We also investigated mitochondrial metabolic factors. mTOR is a regulator of cell growth and metabolism, and is related to mitochondria through translation of nucleus-encoded mitochondrial mRNAs, which increase mitochondrial ATP production. It is also involved in mitochondrial fission depending on the environment. mTOR modulates the cytoskeleton, including microtubule integrity for cell proliferation and migration [25].

Our study demonstrated that phosphorylation of mTOR was significantly increased by treatment with microtubule stabilizers but clearly decreased by treatment with a microtubule disturber. To the best of our knowledge, there is no report regarding the relationship between microtubule stabilizers and mTOR activity. We plan to further investigate the role of microtubule stabilizers and disturbers in relation to phosphorylation of mTOR. We will continue to study microtubule stabilizers for clinical treatment of dysfunctional mitochondria-related diseases such as dystrophy.

## 5. Conclusions

Taken together, our data show that microtubule stabilizers enhance bioenergetic activity of mitochondria and can be potentially used to restore the functional activity of mitochondria in mammalian cells.

## Data Availability

Not applicable.

## Supplementary Materials

The following are available online at https://www.mdpi.com/article/10.3390/cells10123600/s1, Table S1: primer sequences used for real-time qPCR, Video S1: confocal live-cell imaging movie clips of MitoTracker (red)-stained mitochondria in control (A), Taxol-treated (B), EpD-treated (C), and VNB-treated (D) cells.
