# Supplementary material for: Microtubule Integrity Is Associated with the Functional Activity of Mitochondria in HEK293
Source: Cells. 2021 Dec 20;10(12):3600. doi: 10.3390/cells10123600 (PMC8700340; doi:10.3390/cells10123600)
Supplement: Supplementary file 1 [file cells-10-03600-s001.zip › 3 Supplement data_for_HEK293 (2021-10-19) JHL.pptx]

## Slide 1
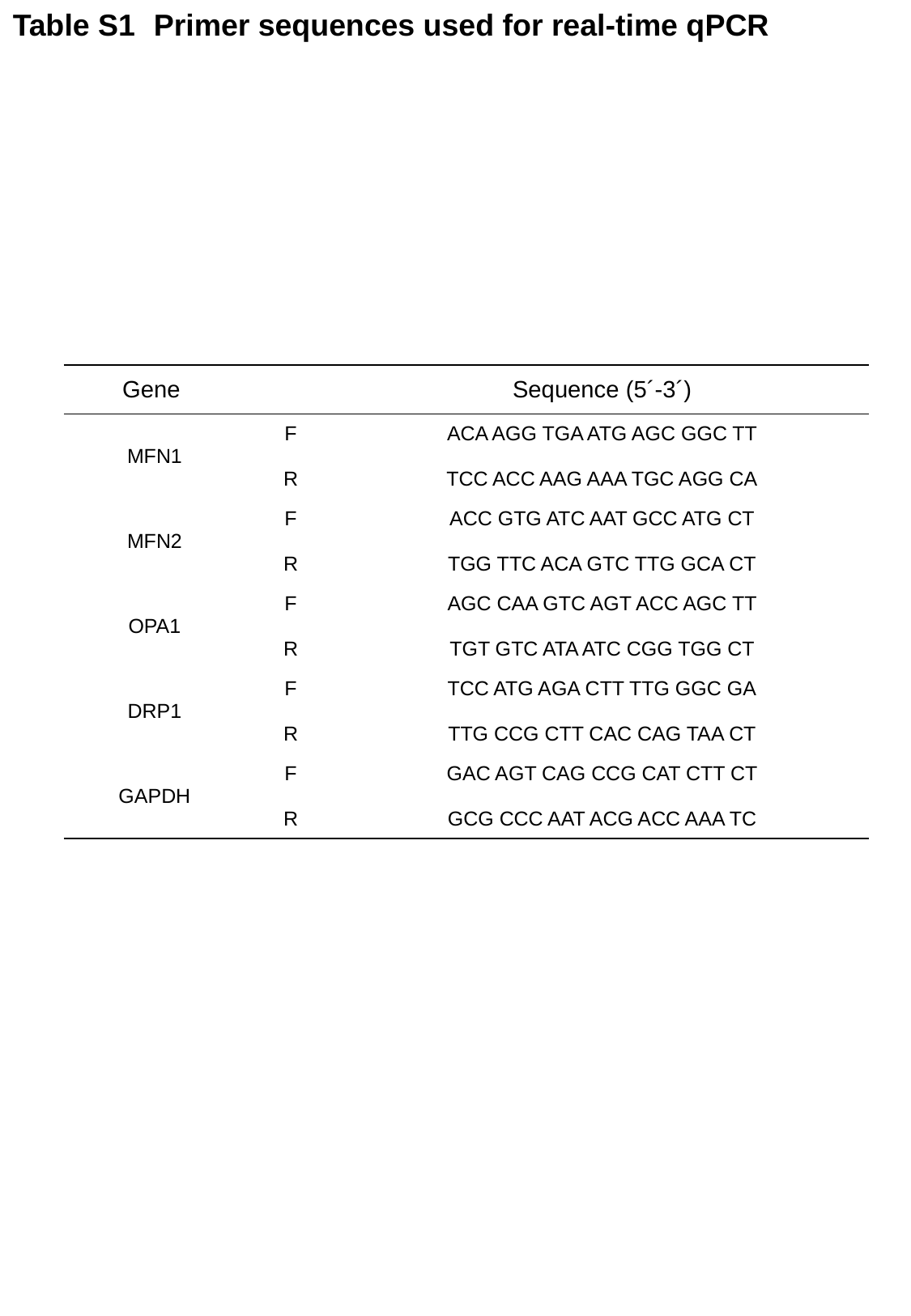

Table S1
Primer sequences used for real-time qPCR
| Gene | | Sequence (5´-3´) |
| --- | --- | --- |
| MFN1 | F R | ACA AGG TGA ATG AGC GGC TT TCC ACC AAG AAA TGC AGG CA |
| MFN2 | F R | ACC GTG ATC AAT GCC ATG CT TGG TTC ACA GTC TTG GCA CT |
| OPA1 | F R | AGC CAA GTC AGT ACC AGC TT TGT GTC ATA ATC CGG TGG CT |
| DRP1 | F R | TCC ATG AGA CTT TTG GGC GA TTG CCG CTT CAC CAG TAA CT |
| GAPDH | F R | GAC AGT CAG CCG CAT CTT CT GCG CCC AAT ACG ACC AAA TC |
